# Performance of Real and Virtual Object Handling Task Between Post-Surgery Wrist Fracture Patients and Healthy Adults

**DOI:** 10.3390/healthcare13121390

**Published:** 2025-06-11

**Authors:** Chun Wei Yew, Kai Way Li, Wen Pei, Mei-Hsuan Wu, Pei Syuan Wu, Lu Peng

**Affiliations:** 1 College of Management, Chung Hua University, Hsinchu 30012, Taiwan; cgh05886@cgh.org.tw (C.W.Y.); kai@chu.edu.tw (K.W.L.); wpei@chu.edu.tw (W.P.); d11303006@chu.edu.tw (P.S.W.); 2 Department of Orthopedics, Hsinchu Cathay General Hospital, Hsinchu 30060, Taiwan; 3 Precision Medicine PhD Program, National Tsing Hua University, Hsinchu 030013, Taiwan; s101082015@gapp.nthu.edu.tw; 4 College of Information Management, Nanjing Agricultural University, Nanjing 210095, China

**Keywords:** distal radius fracture, hand function, human-virtual object interaction, movement time, Fitts’ law

## Abstract

**Background:** Humans interacting with virtual objects is becoming common due to the popularity of the devices adopting the mixed reality (MR) techniques. Assessing hand functions using these devices for medical purposes provides alternatives in addition to the traditional hand function assessment techniques. **Objectives:** The objectives were to compare the movement time (MT) of handing a real and a virtual object between post-surgery wrist fracture patients and healthy adults and to determine the correlation between the MT and commonly adopted hand function indicators. **Methods:** An experiment was performed. A total of 29 participants, including 17 patients and 12 healthy adults, joined. All the participants moved a real or a virtual tube from an origin to a destination. A set of MR device was adopted to generate the virtual object. The MTs were analyzed to compare differences between the patients and the healthy adults. Regression models were developed to predict the MT under experimental conditions. **Results:** The MT of the surgical hand was significantly longer than that of the nonsurgical hand of the patients and was significantly longer than that of the left hand of the healthy adults. The MT was negatively correlated with the commonly adopted hand function indicators, including grip strength, range of motion, hand dexterity score, and Modified Mayo Wrist Score. **Conclusions:** The anticipation that the MT of interacting with virtual objects for patients may reveal hand function characteristics for post-surgery patients was supported. The regression models developed could reveal the progression of hand function recovery for these patients. Having patients interact with virtual objects could be a supplemental approach in assessing their hand functions.

## 1. Introduction

Distal radius fractures are common in the aging population, especially for women over 65, due to osteoporosis risk [1,2,3]. Younger patients may suffer these types of fractures due to slips and falls, automobile accidents, and other upper extremity injury incidences. These fractures may be treated surgically or nonsurgically. Various approaches may be applied in surgical treatments depending on factors, such as the type of fracture, injury severity, joint involvement, displacement, and patient’s age [4]. After a hand surgery, the patient is recommended to go through a rehabilitation program during the recovery period to manage pain and, most importantly, to regain the patient’s hand function, including mobility, strength, and dexterity [5].

Surgeons evaluate treatment effectiveness and disease progression of the patient by diagnosing symptoms of pain and assessing hand functions to monitor the patient’s recovery [6]. These diagnoses and assessments can be performed by measuring data such as range of motion (ROM), grip strength, hand dexterity, and the ability to perform self-care daily living and work activities [5]. Hand function assessment tools such as the Disability of the Arm, Shoulder, and Hand (DASH), the Michigan Hand Questionnaire (MHQ), the Arthritis Hand Function Test [7,8], the Modified Mayo Wrist Score [9,10,11], the visual analogue scale (VAS) of pain [12], and so on, are also commonly used.

In addition to the ROM, grip strength, and hand dexterity, movement time (MT) to complete pointing or object movement tasks may also be applied to examine hand functions and to compare the efficiency of the tasks been performed. This variable has been widely adopted in human–machine interface design [13,14,15,16] and for healthcare and medical purposes [17,18,19]. Fitts’ law has been one of the commonly cited theories in analyzing bodily segment movements and MT. This law claims that the MT of a body segment moving from one location to a target depends on the distance of the movement and the size of the target [20]. An index of difficulty (ID), considering the movement distance (D) and target size (W), was defined to quantify the difficulty of the movement. The following equation explains the Fitts’ law:

MT = *a* + *b* ID (1)

where *a* and *b* are constants, which are determined by experimental data. Mathematically, the ID has no units, and a “bit” is given to quantify the information transfer involved in the movement. It was originally defined as log_2_(2D/W) [20,21,22]. Even though variants, such as log_2_(2D/W + 1) and log_2_(2D/W + 0.5) [23], have been proposed, the original ID definition is still widely used [14,24,25].

Traditionally, Fitts’ law has been applied to analyze the MT for handling real objects or interacting with real targets [13,26,27,28,29,30,31,32]. With the popularity of virtual reality (VR), augmented reality (AR), and mixed reality (MR), this law has also been adopted to analyze the MT of humans interacting with virtual objects in VR/AR/MR environments [25,33,34,35,36] for healthy adults and the general population. VR and AR technologies are increasingly used in rehabilitation for their ability to create interactive therapeutic environments. In physical rehabilitation, patients perform tasks, like reaching, squatting, or stepping, using VR headsets, improving motor skills, strength, and endurance [1]. For neurological recovery, these immersive environments encourage neuroplasticity through controlled, goal-directed movements, aiding motor relearning and cognitive engagement [2,37]. Such approaches enhance traditional rehabilitation, offering innovative ways to improve functional recovery in musculoskeletal and neurological care [1,2,3].

We were curious whether the MT that patients interacting with virtual objects may also reveal hand function characteristics as other commonly adopted hand function data do for post-surgery patients suffering distal radius fractures. We were also interested in establishing a regression model to describe the trend of MT for post-surgery patients in performing real and virtual object movement tasks. Such a model should be capable of revealing the progress of hand function recovery for patients. This study was performed to fulfill these purposes.

## 2. Methods

### 2.1. Human Participants

A total of 29 human participants were recruited and were split into experimental and control groups. In the experimental groups, 17 patients, including 9 females and 8 males, from Hsinchu Cathay General Hospital (HCGH) joined. All patients enrolled in this study were right-handed adults who underwent surgical intervention for distal radius fractures of the left hand. The ages of the male and female participants in this group were 41.8 (±14.4) and 56.0 (±8.1) yrs, respectively. The difference between the two was significant (*p* < 0.05). Exclusion criteria included individuals with significant visual impairments or a prior history of ophthalmological procedures, such as refractive laser surgery for myopia, cataract extraction, glaucoma, or macular surgery. Additionally, participants with a history of wound infections within the preceding three months, epilepsy or seizure episodes, and persistent dizziness or vertigo were also excluded. All patients carried out rehabilitation at home, according to the surgeon’s instructions The patients adhered to medical instructions by performing home-based rehabilitation exercises, comprising active and passive wrist joint mobilizations and stretching, accompanied by heat application. This regimen also included stretching exercises targeting the proximal, intermediate, and distal joints of the fingers, as well as grip-strength training with a squeeze ball for 15 min each, both in the morning and evening. The patients participated in the experiment after at least six weeks of home rehabilitation. Their time after surgery (TAS) ranged from 6 to 113 weeks. They were assessed by a licensed orthopedic surgeon (C.W.Y.) on whether they could complete the tests in this study without difficulty before joining this study.

Upon an outpatient clinic visit, the surgeon assessed the hand function of the patient’s surgery hand using the Modified Mayo Wrist Score (MMWS) and measured the range of motion (ROM) of wrist flexion of this hand. The patient also responded to the VAS of pain on his/her surgery hand in a range from 0 to 100. In addition, the grip strength of the patient was measured using a dynamometer (Takei, T.K.K.5825, Takei Scientific Instruments, Tokyo, Japan) under an elbow flexed at 90° and at a grip span of 5 cm. The participant then joined a pegboard test (PT) for hand dexterity measurements [38]. The number of pins successfully inserted into the slots within 30 s was recorded. The grip strength and PT score were collected for each of the two hands.

In the control group, 12 healthy adults, including 5 males and 7 females, joined. These participants were recruited from the staff from the institute where the first author served (CHU and HCGH). All participants were right-handed adults, without history of prior hand surgery. The ages of the male and female participants in this group were 50.4 (±7.0) and 51.1 (1.1) yrs, respectively. The difference between the two was not significant. The exclusion criteria applied to this group were identical to those of the experimental group. They did not go through the clinic diagnosis of hand function as the participants in the experimental group did. However, their grip strength and hand dexterity were also measured. The age and hand function data of all the participants are shown in Table 1. All the participants were right-handed. None of the participants have prior experience of using an AR/MR device.

In total, 17 patients and 12 healthy adults joined, and a total of 29 participants were tested. They comprised a convenient sample. A post hoc power analysis was conducted using G*Power 3.1.9.7 [39] for the repeated between-subjects design, yielding an achieved power of 0.95 (*f* = 0.50, *N* = 29, *α* = 0.05). This value exceeds the conventional threshold of 0.80 [40], indicating that the study had sufficient power to detect medium to large effects.

### 2.2. Tube Movement Experiment

**Figure 1:**
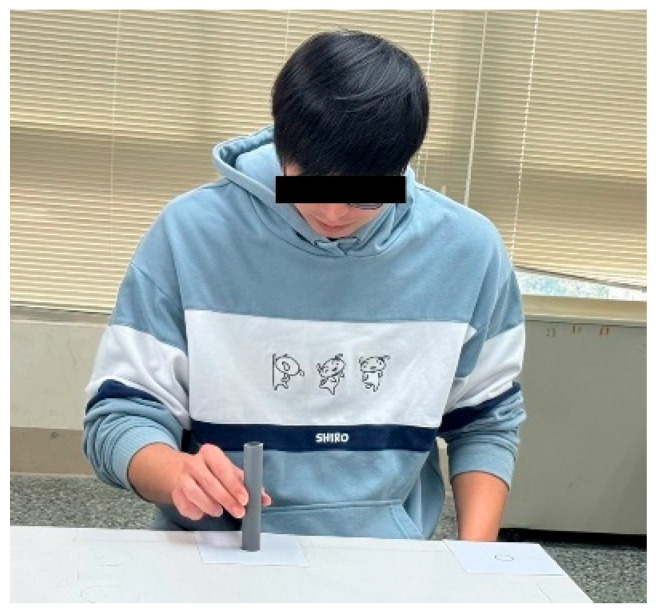
Moving a tube from one location to another in lateral direction.

All the participants joined a tube movement experiment. An experimenter explained the objectives and procedure of the study to the participants. All the participants read and signed an informed consent. The participant sat approximately 25 cm in front of a table 75 cm high and pinched a vertically positioned tube from a starting point and moved it and put it into a destination (see Figure 1). Two circles were marked on the table to indicate the location of the starting point and the destination. The size (W), or the diameter, of these circles was 2.2 cm. Four distances between the centers of the two circles were tested, as follows: 12.5, 25, 37.5, and 50 cm. The ID (or log_2_(2D/W)) comprised by these distances and W ranged between 3.5 and 5.5 bit. Both the right and left hands of the participants were tested. The illuminance on the table, measured using a light meter (Lux Meter, Trans Instrument, Singapore), was 735 (±79) lx.

**Figure 2:**
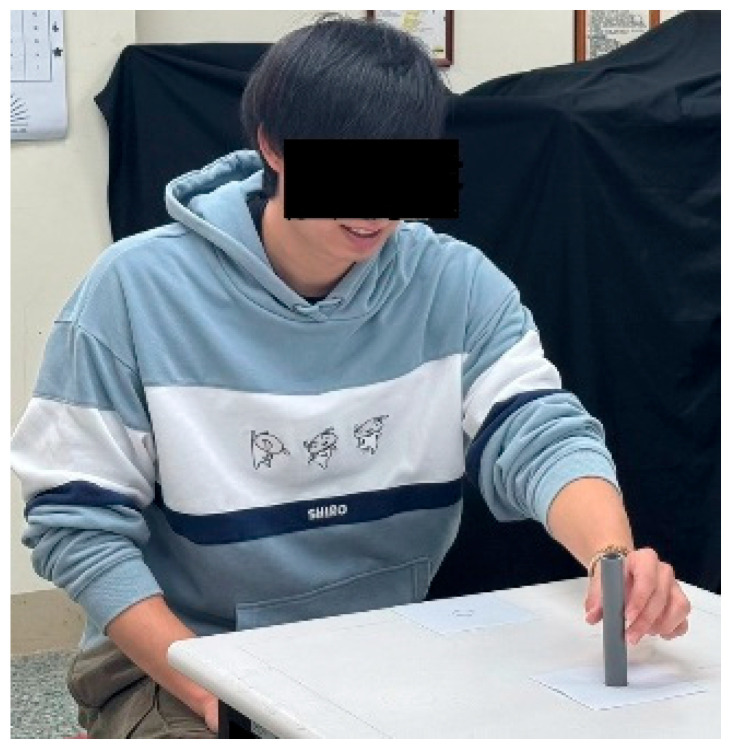
Moving a tube from one location to another in anterior–posterior direction.

The tube movement trials were carried out in both lateral and anterior–posterior directions. For the lateral movement, the circles were marked along the table approximately 5 cm from the edge (see Figure 1). For the anterior–posterior movement, a circle was marked approximately 5 cm from the edge of the table, and another circle was marked at a distance away from the table edge (see Figure 2). In each trial, when one of the circles was adopted as the starting point, the other was then the destination. In other words, two trials were performed for each of the movement directions, where each circle was adopted as the starting point once.

**Figure 3:**
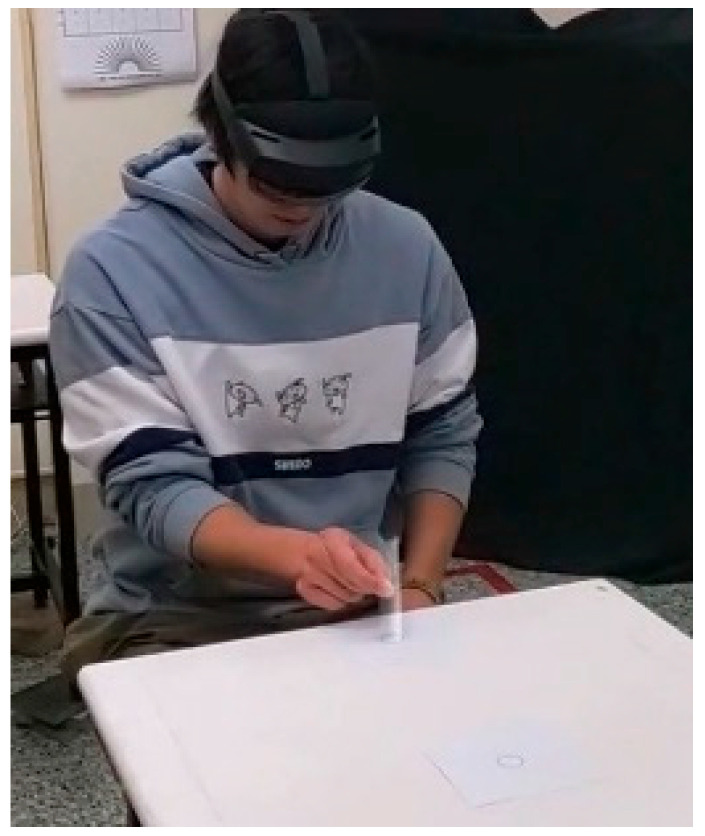
Handling a virtual tube.

There were two types of tubes—real and virtual. For real tube, a 12.5 cm grey plastic tube with a diameter of 2.2 cm was adopted (see Figure 1 and Figure 2). This tube has a mass of 30 g. For the virtual tube, a virtual tube of the same dimensions and color as the real one was generated using a Holopipe app in a Microsoft Hololens 2 MR device (Microsoft^®^, Redmond, WA, USA). Before the trial of moving the virtual tube, the participants were instructed on how to interact with the virtual tube when wearing the Hololens 2 and had the opportunity to practice until they were familiar with manipulating the virtual tube. Figure 3 shows a trial handling a virtual tube when wearing an MR device.

### 2.3. Experiment Design and Data Analysis

The tube movement time (MT) was the dependent variable of the experiment. This time was measured starting from hand–tube contact at the starting point until hand–tube separation at the destination and was extracted from the timer of the videos for each trial. For virtual tube movement, the video was captured using the built-in cameras in the Hololens 2 and was shared to a laptop computer for later processing. For real tube movement, the video was taken using a smartphone (iPhone 12, Apple Inc., Cupertino, CA, USA) and then uploaded to a laptop computer. For each participant, there were 64 experimental conditions (2 types of tube × 2 movement directions × 2 hands × 2 starting points × 4 distances). There was a scheduled break for approximately five minutes when the participant completed half (32) of the trials. In addition to this scheduled break, the participant could request to take a break any time when he or she had such a need. The order of these conditions for each participant was randomly arranged. Descriptive statistics and Satterthwaite *t*-test were performed. The Satterthwaite *t*-test was employed when our data exhibited unequal variances between groups and included unbalanced sample sizes. Analysis of variance (ANOVA), correlation, and regression analysis were also performed. These statistics analyses were conducted using the SAS 9.0 software (SAS^®^ Institute, Cary, NC, USA). An online effect size calculator [41] was adopted, because the SAS does not calculate the effect size directly.

## 3. Results

The results of a Satterthwaite *t*-test [42] indicated that the age between the experimental and control groups was not significantly different. The grip strength of the left hand of the experimental group (19.8 ± 9.5 kgf) was significantly (*p* < 0.05) lower than that of the control group (27.4 ± 8.3 kgf). The grip strength of the right hand of the experimental group (28.1 ± 8.8 kgf) was not significantly different from that of the control group (27.6 ± 8.7 kgf). The PT score between the left hand of the experimental group (13.2 ± 2.0) was not significantly different from that of the control group (14.0 ± 2.0). Neither was this score of the right hand between the two groups (13.9 ± 2.0 vs. 14.9 ± 1.7) significant.

**Figure 4:**
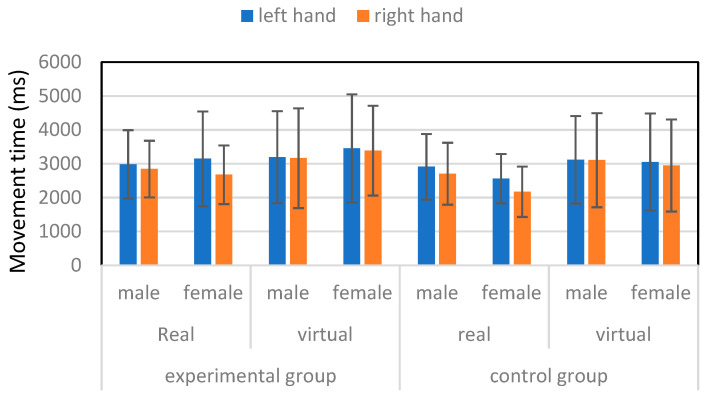
Movement time for male and female participants in experimental and control groups.

Figure 4 shows the MT averaged over all starting points and moving distances for male and female participants in experimental and control groups handling real and virtual tube. A Satterthwaite *t*-test results indicated that the overall MT of the experimental group (3120.8 ± 1297.8 ms) was significantly (*p* < 0.0001) higher than that of the control group (2822.5 ± 1174.4 ms). The 95% confidence interval (CI) and Cohen’s D of the difference of these two groups were (185.5, 410.9) ms and 0.24, respectively. Satterthwaite *t*-test results indicated that the MT of the left hand of the participants in the experimental group (3219.6 ± 1396.9 ms) was significantly (*p* < 0.001) higher than the MT of the left hand of the participants (2910.7 ± 1156.4 ms) in the control group. The 95% CI and Cohen’s D of the difference of these two groups were (144.3, 473.3) ms and 0.24, respectively. Similar results were also found when comparing the MT of the right hand between the experimental (3021.9 ± 1184.0 ms) and control groups (2734.3 ± 1186.7 ms) that the MT of the former was significantly (*p* < 0.0001) higher than the latter. The 95% CI and Cohen’s D of the difference of these two groups were (134.1, 441.2) ms and 0.24, respectively.

An ANOVA was performed for the experimental group data to analyze the effects of sex, type of tube, moving direction, hand, and moving distance on the MT. The results showed that the effects of type of tube (*p* < 0.0001), hand (*p* < 0.05), moving direction (*p* < 0.001), and moving distance (*p* < 0.0001) were significant on the MT, while the effects of the sex were insignificant. The Duncan’s multiple range test results showed that the MT moving a virtual tube (3328.9 ± 1447.4 ms) was significantly longer than that of handling a real tube (2912.6 ± 1091.4 ms). The 95% CI and Cohen’s D of the difference of these two types of tube were (241.9, 590.8) ms and 0.32, respectively. Moving a tube in the anterior–posterior direction (3282.2 ± 1474.7 ms) was significantly longer than that of moving a tube in the lateral direction (2959.4 ± 1070.5 ms). The 95% CI and Cohen’s D of the difference of these two directions were (147.5, 498.2) ms and 0.25, respectively. The MT of the surgical left hand (3219.6 ± 1396.9 ms) was significantly longer than that of the nonsurgical right hand (3021.9 ± 1184.0 ms). The 95% CI and Cohen’s D of the difference of these two hands were (21.4, 373.8) ms and 0.15, respectively. The MT of moving a tube 50 cm (3592.8 ± 1603.3 ms) was significantly longer than those of moving 37.5 cm (3331.9 ± 1247.8 ms), 25 cm (2959.6 ± 1141.2 ms), and 12.5 cm (2598.8 ± 873.9 ms). The MT of moving a tube 37.5 cm was significantly longer than those of moving 25 cm and 12.5 cm. The difference between moving 25 cm and 12.5 cm was also significant.

An ANOVA was also performed for the control group on the MT. The results showed that the effects of sex (*p* < 0.0001), type of tube (*p* < 0.0001), hand (*p* < 0.05), moving direction (*p* < 0.01), and moving distance (*p* < 0.0001) were significant on the MT. The Duncan’s multiple range test results showed that the MT of male participants (2960.7 ± 1168.1 ms) was significantly longer than that of their female counterparts (2684.4 ± 1165.5 ms). The 95% CI and Cohen’s D of the difference of these two genders were (133.2, 419.4) ms and 0.24, respectively. Moving a virtual tube (3056.7 ± 1367.5 ms) was significantly longer than that of handling a real tube (2588.3 ± 883.9 ms). The 95% CI and Cohen’s D of the difference of these two types of tube were (327.2.9, 609.6) ms and 0.41, respectively. Moving a tube in the anterior–posterior direction (2918.0 ± 1233.7 ms) was significantly longer than that of moving a tube in the lateral direction (2727.1 ± 1104.9 ms). The 95% CI and Cohen’s D of the difference of these two directions were (47.3, 334.6) ms and 0.16, respectively. The MT of the left hand (2910.7 ± 1156.4 ms) was significantly longer than that of the right hand (2734.3 ± 1186.7 ms). The 95% CI and Cohen’s D of the difference of these two hands were (32.7, 320.1) ms and 0.15, respectively. The MT of moving a tube 50 cm (3133.8 ± 1315.1 ms) was significantly longer than those of moving 37.5 cm (2914.0 ± 1060.2 ms), 25 cm (2741.4 ± 1082.7 ms), and 12.5 cm (2500.9 ± 1136.4 ms). Both the MT of moving a tube 37.5 cm and 25 cm were significantly longer than that of moving 12.5 cm. The difference between moving 37.5 cm and 25 cm was insignificant.

The Pearson’s correlation coefficients between MT and age and between MT and TAS for the experimental group were 0.16 (*p* < 0.0001) and −0.24 (*p* < 0.0001), respectively. The Pearson’s correlation coefficient between MT and age for the control group was 0.13 (*p* < 0.0001). Pearson’s correlation coefficients between MT and each hand function-related variables for the experimental group are shown in Table 2.

Equations (2) and (3) were established to predict the MT, based on the experimental and control group data, respectively:

(2) $$\mathrm{MT}=431.61\;\times \;\mathrm{type}+338.07\;\times \;\mathrm{direction}+5145.88\;\times \;\frac{hand}{TAS}+558.00\;\times \;\mathrm{ID}$$

MT = 515.13 × type + 237.65 × direction + 223.09 × hand + 496.76 × ID(3)

where TAS is the time after surgical (week), and type, direction, and hand are dummy variables, as follows:

             type = 0 for real tube

                     =1 for virtual tube

             direction = 0 for lateral movement

                             =1 for anterior-posterior movement

             hand = 0 for right, nonsurgical hand

                       =1 for left, surgical hand

             ID = log_2_(2 × distance/W)

where W is 2.2 which was the size of the target in centimeter. The regression coefficients in Equation (2) were all significant at *p* < 0.0001. The regression coefficients in Equation (3) were all significant at *p* < 0.0001 except for the direction (*p* < 0.001) and hand (*p* < 0.01). The adjusted R^2^ of these two equations were 0.87 and 0.86, respectively.

## 4. Discussion

Having post-surgery patients interact with virtual objects to observe their hand functions is an innovative attempt compared to the traditional approaches. All the participants completed the trials without difficulty. The grip strength data show that the left, surgical hand of the experimental group was significantly lower than that of the control group, while the grip strength of the right hand of the experimental group was not significantly different from that of the control group. This revealed that the grip strength of the patients’ surgical hands has recovered partially and was inferior to that of their healthy adult counterparts. The PT data, however, showed that the hand dexterity of the surgical hand of the patients was not inferior to that of the healthy adults in the control group.

Human interaction with virtual objects is surely different from that with real ones. Many factors could contribute to the variations of the MT of hand movement under real and virtual environments. Tactile feedback, for example, could facilitate hand movement involving target positioning and lead to less MT. This was verified in Zhao et al. [25] when they requested their participants to point real and virtual targets and compared the MT between the two. Li and Nguyen [35], however, found that transferring a virtual object was more efficient than transferring a real one. They argued that a virtual object was weightless and was easier to manipulate than a real one. Our results were consistent with those in the former [25] and were inconsistent to the latter [35]. It seemed that the weight of the object, in our study, was not a factor affecting the MT. Even if we did not measure the pinch force required to handle the real tube, this force was probably quite small because the real tube has a mass of only 30 g. Handling a real tube was more intuitive than handling a virtual one. Our participants seemed to have more difficulty in handling a virtual tube than a real one, especially when they were positioning and placing it into the circle, probably also due to the lack of tactile sensation.

Our findings showed that moving a tube in the anterior–posterior direction was significantly longer than that of moving in the lateral direction. This movement efficiency was attributed to the involvement of upper extremity joints. Hand movement in the lateral direction involves primarily the movement of the elbow joint, while both the elbow and shoulder joint movement are required in the anterior–posterior movement. The latter was, therefore, less efficient than the former and thus required more time to complete. These findings were consistent with those in the literature [30]. Movement direction affects the MT, which has also been reported in the lower extremity in the literature [31,32].

The Pearson’s correlation coefficients between MT and TAS and hand function variables were all negative. This was reasonable, as longer TAS and better hand functions should lead to shorter MT. The correlation coefficients of the MT of the surgical hand handling the virtual tube between the MMWS (−0.46, *p* < 0.0001) and between the grip strength (−0.42, *p* < 0.0001) were relatively higher than those in handling the real tube (−0.19, *p* < 0.05 and −0.18, *p* < 0.01, respectively) implying that the MT could reveal hand function characteristics more consistently in the virtual condition than those in the real condition.

Equation (2) was established using the data of the experimental group. It may be applied to similar groups suffering a distal radius fracture surgery on the left hand with a TAS between 6 to 113 weeks. The R_adj_^2^ of Equation (2) is 0.87, indicating that 87% of the variation of the MT may be explained by this equation. The regression coefficient indicates the change of the dependent variable due to the change of the independent variable for one unit. The regression coefficient in Equation (2) indicates that, on average, handling a virtual tube required 431.61 ms more than handling a real tube. Handling a tube in the anterior–posterior direction, on average, needs 338.07 ms more than handling a tube in the lateral direction. Using a left surgical hand to relocate the tube requires 5145.88/TAS (ms) more than using a right nonsurgical hand. This also implies that MT is inversely dependent on TAS. Based on this equation, the MT of the surgical hand for patients with a TAS of 6 weeks is, on average, 857.65 ms more than their right nonsurgical hand. For patients with a TAS of 113 weeks, the difference of the MT between the left surgical and right nonsurgical hands diminished to 45.54 ms only. The trend of MT decreasing over TAS indicates the recovery of the hand function of the surgical hand. There was no TAS term in Equation (3), because it was generated using the data from the healthy adults in the control group.

The inverse of the regression coefficient for ID reflects the information processing rate of hand movements [20,21,22,23]. Previous studies have demonstrated that the information processing rate for arm movements is approximately 10 bits per second [43,44]. This information processing rate was, however, obtained under hand–real object/target interaction conditions only under different experimental scenarios that may not be applicable to our study. Li and Nguyen [35], on the other hand, have found their rate ranged between 1.7 to 3.4 bits per second when handling a virtual object under different experimental conditions. The information processing rates for handling the tube predicted by Equations (2) and (3) were 1.8 and 2.0 bits per second, respectively. These rates are surely quite low compared to the findings in earlier studies [43,44] but are within the range in Li and Nguyen [35] even though the participants in the two studies were in different age groups (50 s vs. 20 s). Both our data and those in Li and Nguyen [35] support that the information processing rate in handling a tube-shaped object is much lower as compared to the ordinary arm movement tasks reported in the literature [43,44].

There are limitations to this study. The first one was that the range of ID (3.5 to 5.5 bits) was relatively small. This was due to the space constraint of moving the bar in front of the body of the participant using one hand. This may hamper the generalization of our findings and application of our regression models. The ID in the original pin transfer experiment in Fitts [20] ranged from 3 to 10 bits. A wider range of ID implies a broader hand movement condition is covered. Future research may be designed to expand the range of the ID, especially for the surgical hand–virtual object interactions. This implies scenarios with more object sizes and more moving distance should be tested. The second limitation was that all the participants were right-handed, and all the patients suffered distal radius fractures on their left hands. This inclusion decision was made to avoid the confounding effects of surgical–nonsurgical and dominant–nondominant hand. Our data, apparently, cannot be used to interpret the hand function problems for patients suffering distal radius fractures on their right or dominant hands. The third limitation was that our virtual object movement trials were realized via the Holopipe application in the Hololens 2 MR device. Our findings may need to be validated using other applications and MR devices.

The implication of our findings is that hand function assessment may also be performed by utilizing MR devices by having patients interact with virtual objects. A surgeon needs various tools to assess the hand functions of the patients objectively. For example, a dynamometer is needed to measure grip strength, and a PT test kit is needed to measure hand dexterity. With proper application designs, there is an opportunity that a surgeon may conduct different hand function tests using simply one MR device. Such designs may allow the documentation of the assessment results and thus improve the efficiency of clinical assessment of patients’ hand functions. To achieve this purpose, more hand function assessment MR applications will be required. In addition, the disadvantages of using an MR device are that the device is costly, and most physicians are not familiar with such advanced devices. Using an MR device to assist in diagnosing the hand functions of patients will not be common before these problems are solved.

## 5. Conclusions

Our data showed that the MT of the experimental group was significantly longer than that of the control group. The MT of the left surgical hand of the patients was significantly longer than that of the right nonsurgical hand. This MT was also significantly longer than that of the left hand of the healthy participants in the control group. These findings, together with our correlation analyses results, support our anticipation that the MT of interacting with virtual objects for patients may reveal hand function characteristics for post-surgery patients suffering distal radius fractures. Our regression model established using patients’ data indicates that the MT is inversely dependent on the TAS. It could reveal the progression of hand function recovery for patients. Having patients interacting with virtual objects could be a supplemental approach in assessing their hand functions.

## Figures and Tables

**Table 1:** Hand function data of the participants.

	Experimental	Group	Control	Group
	Male (n = 8)	Female (n = 9)	Male (n = 5)	Female (n = 7)
TAS (weeks)	46.4 (38.5)	30.3 (24.3)	-	-
MMWS-L	70.6 (8.2)	69.4 (12.1)	-	-
VAS-L	23.1 (2.6)	22.2 (3.6)	-	-
ROM (°)-L	11.9 (3.7)	13.9 (5.5)	-	-
GS (kgf)-L *	25.4 (10.1)	14.9 (5.7)	35.6 (6.0)	21.6 (2.4)
GS (kgf)-R **	34.9 (8.6)	22.2 (2.3)	36.4 (5.6)	21.3 (2.7)
PT-L	13.3 (1.4)	13.2 (2.6)	13.4 (1.1)	14.4 (2.4)
PT-R	13.5 (1.5)	14.2 (2.4)	13.8 (1.9)	15.6 (1.3)

Note: TAS: time after surgery; MMWS: Modified Mayo Wrist Score; MMWS, ROM, and VAS applied to surgical hand only. GS: grip strength; L: left hand; R: right hand; * *p* < 0.05 and ** *p* < 0.01 for between sex comparison; Cohen’s Ds for GS for L and R comparing males and females within experimental group are 1.27 and 2.03, respectively.

**Table 2:** Pearson’s correlation coefficients between MT and each of hand function and diagnostic data of the surgical hand for the experimental group.

	Virtual Tube		Real Tube	
	Left Hand	Right Hand	Left Hand	Right Hand
Grip strength (kgf)	−0.42 ***	−0.19 **	−0.18 **	−0.18 **
PT score	−0.15 *	−0.14 *	−0.18 **	−0.05
MMWS	−0.46 ***	-	−0.19 *	-
ROM	−0.27 ***	-	−0.27 ***	-
TAS	−0.22 *	-	−0.33 ***	-

TAS: time after surgery. * *p* < 0.05, ** *p* < 0.01, *** *p* < 0.0001.

## Data Availability

Data will be available upon request to the corresponding author.
